# AI-based wearable system for fall risk prediction in older adults using sEMG and plantar pressure data

**DOI:** 10.3389/fbioe.2026.1698253

**Published:** 2026-04-30

**Authors:** Xikun Ma, Yuandan Liao, Gang Tan, Boquan Qin, Guohui Chen, Xi Liu, Peifang Li, Jiali Chen, Xiao Ke, Hui Zhang

**Affiliations:** 1 Department of Orthopedic Surgery and Orthopedic Research Institute, West China Hospital, Sichuan University, Chengdu, Sichuan, China; 2 Med-X Center for Manufacturing, Sichuan University, Chengdu, Sichuan, China; 3 Department of Orthopedics, West China School of Public Health and West China Fourth Hospital, Sichuan University, Chengdu, Sichuan, China; 4 School of Artificial Intelligence, Shenzhen Technology University, Shenzhen, China

**Keywords:** AI-based wearable system, fall risk assessment, machine learning, plantar pressure analysis, surface electromyography

## Abstract

Falls among older adults pose a major healthcare and social burden, making early identification of high-risk individuals essential for prevention. This study presents a portable, non-invasive AI-based wearable system that predicts fall risk using surface electromyography (sEMG) and plantar-pressure measurements collected during overground walking. sEMG electrodes were placed bilaterally over eight key lower-limb muscles—tibialis anterior, peroneus longus, medial and lateral gastrocnemius, rectus femoris, vastus medialis, vastus lateralis, and biceps femoris—while pressure insoles captured loading at eight anatomical foot regions. Ninety-four older adults (mean age 69.6 ± 10.0 years; 57 females), including 57 non-fallers and 37 individuals who met ICD-10 diagnostic criteria for “propensity to fall,” participated in the modeling study. The signals from both devices were streamed wirelessly to a central acquisition unit for synchronized processing. Extracted features included muscle activation contribution, mean frequency, mean power frequency, and cumulative plantar-pressure impulses. These features served as model input. To reduce data dimensionality, Principal Component Analysis (PCA) and Linear Discriminant Analysis (LDA) were applied. PCA retained a variance structure, whereas LDA maximized class separability. Three machine-learning classifiers—Support Vector Machine (SVM), Random Forest (RF), and Extreme Gradient Boosting (XGB)—were trained using Leave-One-Out Cross-Validation. LDA substantially improved performance across all models, with LDA + SVM achieving the highest accuracy (0.88), precision (0.92), recall (0.85), and F1-score (0.87). An independent clinical validation study involving ten additional older adults demonstrated that LDA-based models generalized well beyond the original dataset. Compared with existing fall-detection or multimodal EMG-based systems that focus on simulated falls, young participants, or non-portable laboratory equipment, the proposed framework enables physiologically interpretable, clinically deployable fall-risk prediction during natural gait. These findings highlight the promise of dual-modality wearable sensing for proactive fall prevention in geriatric populations.

## 1 Introduction

The challenges posed by population aging are a growing concern in many countries, leading to significant health and social implications (Tu et al., 2022; Jakovljevic et al., 2023). Among the elderly population, falls are one of the leading causes of injury, disability, and mortality, with severe consequences for those affected (Gillespie et al., 2009). The underlying causes of falls are multifaceted and typically fall into three broad categories: environmental, behavioral, and health-related factors (Mohan et al., 2024). Among these, health-related factors such as muscle strength, particularly in the lower limbs, and gait stability have been identified as significant contributors to fall risk (Simpkins and Yang, 2022; Nascimento et al., 2022). Previous studies have highlighted that muscular atrophy and gait imbalance are key determinants of increased risk of falling among older adults (Sorock and Labiner, 1992; Menant et al., 2012).

Physiological electrical signals are commonly used to monitor fall risks, playing a crucial role in supporting the wellbeing of aging populations. Surface electromyography (sEMG) is a widely recognized technique to assess muscle activity and function by extracting characteristics from the electrophysiological signals of muscles. sEMG has been used in clinical settings to evaluate muscle health and function (Arjunan et al., 2014; Naik et al., 2008; Wei et al., 2009; Chowdhury et al., 2013). In the literature, Cheng et al. (2013) proposed using sEMG in combination with accelerometers for fall detection. Boccia et al. (2015) demonstrated its utility in monitoring age-related changes in the neuromuscular system, thus helping identify the risks of falls. A 2017 study (Xi et al., 2017) used sEMG detection methods to analyze compensatory balance responses in normal populations aged 18–40 years under sudden external forces, assessing the risk of falling in such scenarios. On the other hand, the plantar pressure signal refers to the data obtained by measuring the pressure exerted on the soles of the feet during various activities, such as walking, running, or standing. It has been used to assess gait stability by examining the distribution of the pressure collected (Mickle et al., 2011). Research has shown that community-dwelling fallers exhibit significantly higher peak pressure and integral pressure time during gait under the feet compared to non-fallers, increasing their risk of falls (Mickle et al., 2011). Both sEMG and plantar pressure data offer direct assessments of an individual’s physiological function and are easily collected using affordable, portable, and non-invasive sensors (Xi et al., 2017; Georgiou and Koutsos, 2018).

Recent research has primarily focused on early-stage fall detection for elderly individuals living independently, aiming to identify fall events promptly and alert caregivers or emergency services to minimize adverse outcomes (Xi et al., 2017; Liu et al., 2019; Torres-Guzman et al., 2023; Tewari et al., 2023; Brandstötter et al., 2023; Xu et al., 2022). However, these warning mechanisms are activated only after a fall has occurred, and the resulting injuries—such as fractures—are often irreversible. A proactive strategy that identifies individuals at high risk before a fall happens is therefore more desirable. Prior studies have frequently relied on single-source electrical signals (Rosenblum et al., 2022; Veiskarami et al., 2022), which provide only a narrow perspective on the complex neuromuscular and biomechanical factors contributing to fall risk. Such single-modality approaches, often based on statistical comparisons under specific laboratory conditions, offer limited value for robust clinical risk assessment.

Beyond these single-modality studies, a substantial body of literature has explored wearable-sensor–based fall detection and fall-risk assessment using machine-learning techniques. Early work by Albert et al. (2012) demonstrated that mobile-phone accelerometers could accurately classify multiple fall types, establishing the feasibility of inertial-sensor–driven fall monitoring. Building upon this foundation, Ozdemir and Barshan (2014) systematically evaluated machine-learning algorithms with body-worn inertial sensors for activity recognition and fall detection, showing that rich time-series features substantially improve classification performance. As a high-risk group for falls, older adults exhibit significant differences from younger, active populations in terms of muscle strength, reaction capability, balance capacity, and nutritional status. Consequently, data trained on younger cohorts may not adequately represent real-world application scenarios involving the elderly. More recently, Howcroft et al. (2017) developed prospective fall-risk prediction models for older adults using pressure-sensing insoles and multi-location accelerometers, highlighting the potential of multi-sensor gait signatures for clinically meaningful risk stratification. Nevertheless, most of these systems rely primarily on inertial data and focus on detecting fall events rather than proactively assessing fall risk, and they do not incorporate neuromuscular information such as sEMG.

In parallel with inertial-sensor research, several multimodal wearable systems have integrated sEMG with additional biomechanical or physiological signals to improve fall-related analysis. Xi et al. (2020) combined four-channel lower-limb sEMG with three plantar-pressure channels to classify postures, gait activities, and experimentally induced falls in twelve healthy young adults, achieving sensitivities and specificities exceeding 96%. Cheng et al. (2013) fused lower-limb sEMG with trunk and thigh accelerometers, using histogram-based segmentation and double-stream HMMs to recognize daily activities and detect simulated falls with over 98% accuracy in ten healthy adults. Siwadamrongpong et al. (2022) further explored EMG–IMU fusion in a prototype system for real-time fall detection and prediction, although its evaluation was limited to a single young participant, restricting generalizability. Complementing these efforts, da Silva et al. (2024) examined fall risk in thirty older adults using sEMG and dynamometry during maximal isometric contractions; however, the method requires non-wearable laboratory equipment and does not capture gait or plantar-loading behavior.

Taken together, existing multimodal EMG-based studies either focus on fall-event detection in controlled laboratory environments, involve small samples of young adults, or rely on non-portable devices that are unsuitable for routine screening. As a result, portable systems capable of jointly capturing multi-channel sEMG and plantar-pressure signals during natural walking in older adults—and validated within clinical workflows—remain scarce, highlighting a critical gap addressed by the present work.

**FIGURE 1:**
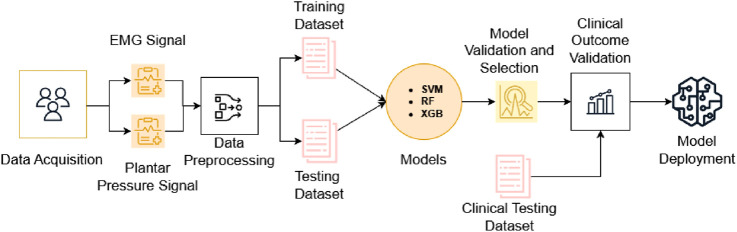
Flowchart illustrating the prediction and validation steps of the proposed framework.

In this study, we propose a novel AI-based wearable system aimed at monitoring and predicting the risk of falls in the elderly. The system utilizes well-designed sensors to collect sEMG and plantar pressure data, from which several features are extracted. A new analytical framework is introduced to process these features and build predictive models of fall risk using machine learning algorithms, including support vector machine (SVM), Random Forest (RF), and extreme gradient boost (XGB) with dimension reduction techniques. After that, an independent clinical dataset is used to validate the prediction model. The overall workflow of the proposed prediction and validation framework is shown in Figure 1. The results demonstrate the potential of this system for clinical application. Integrating this framework into clinical workflows can help physicians identify high-risk individuals and implement targeted interventions, reducing the incidence and impact of falls in aging populations.

## 2 Data collection

### 2.1 Participants

Eligible participants were required to meet the following criteria: age ≥ 60 years, ability to use wearable devices provided by the research team, and independent completion of device operation and walking tests. Individuals were excluded if they had severe lower limb deformities or joint mobility limitations, severe muscle weakness secondary to immune disorders or other pathologies affecting ambulation, severe central nervous system disorders (e.g., prior cerebral hemorrhage or infarction) that contributed to gait dysfunction, or dependence on assistive devices/long-term bedridden status due to comorbidities. Based on questionnaire assessments, participants were classified into two groups: Class 0 (n = 57) comprised individuals with no history of falls, while Class 1 (n = 37) included those with documented falls, all fulfilling the diagnostic criteria for the “propensity to fall” (ICD-10 code R29.6) according to the 1992 International Classification of Diseases, 10th revision (ICD-10) guidelines established by the World Health Organization (WHO).

### 2.2 Sensors and recording acquisition

**FIGURE 2:**
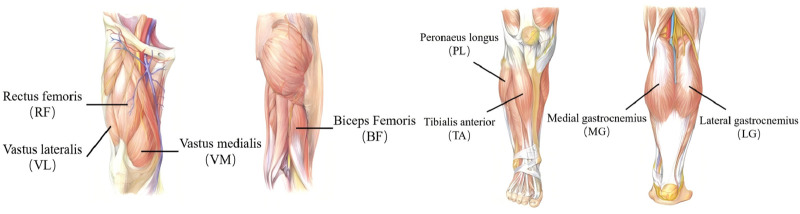
sEMG electrode placement on lower limb muscles.

sEMG signals from the lower limbs were collected using the JE-TB0820 sEMG device (Anhui Aili Intelligent Technology Co., Ltd.), which includes an 8-channel system, a data collector, and a surface EMG acquisition and analysis platform. The signals were sampled at a rate of 2000 Hz. An eight channel bandpass filter (10–500 Hz) isolates relevant signal frequencies, followed by full-wave rectification to produce a unidirectional output. The following muscles were selected for the collection of sEMG: anterior tibialis (TA), medial gastrocnemius (MG), lateral gastrocnemius (LG) from the calf and rectus femoris vastus lateralis (VL) and biceps femoris (BF), semitendinosus (ST) and vastus medialis (VM) from the thighs (see Figure 2). The selection of these muscles is based on the relevant literature (Tong et al., 2014; Su et al., 2017) and suggestions from physicians. The collection process involved applying disposable electrodes to the eight muscles on each leg, with two electrodes aligned along the muscle’s long axis and a third positioned to form an isosceles triangle to minimize interference. Dual-channel sEMG cables were connected to the electrodes and device, ensuring proper metal contact, with the cables arranged to prevent signal disruption.

**FIGURE 3:**
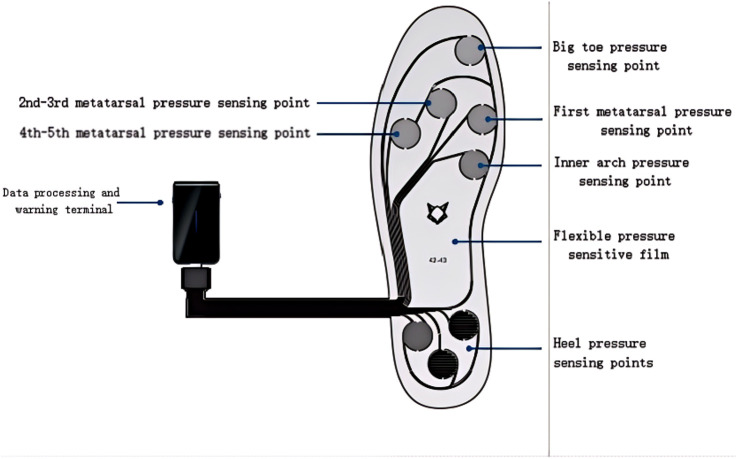
Schematic of plantar electronic skin system.

Plantar pressure signals were collected from the Yuezu plantar electronic skin (pressure insole system), developed by Cyberpi (Wuxi) Technology Co., Ltd (see Figure 3). The system consisted of five pairs of electronic plantar skins (sizes: 36–37, 38–39, 40–41, 42–43, 44–45), two data processing and warning units, and two charging cables. The plantar electronic skin enabled dynamic, real-time collection and transmission of raw plantar pressure signals at a sampling frequency of 100 Hz. The plantar pressure zones included hallux, first metatarsal, second to third metatarsals, fourth to fifth metatarsals, medial arch, and heel (medial, posterior, and lateral sections).

## 3 Data analysis

### 3.1 Feature extraction and selection

Basic information such as age, sex, height, weight, and BMI is selected in the prediction framework. These features can capture individual variation of participants and hence improve the model accuracy. The description of the basic information is shown in Table A1 in the Appendix. Features extracted from sEMG signals including the relative contribution rate (i.e., contribution), the mean frequency (MF) and the mean power frequency (MPF). The time-normalized integrated sEMG of each muscle was calculated as the average rectified sEMG amplitude over the walking trial.

$${ARVE}_{m}=\frac{1}{N_{m}}\overset{N_{m}}{\underset{i=1}{\sum }}\left|sEMG_{m}\left(i\right)\right|,$$ (1a)

$${Contrib}_{m}=\frac{{ARVE}_{m}}{\sum_{i=1}^{16}{ARVE}_{i}}\times 100\%.$$ (1b)

where $sEMG_{m}\left(i\right)$ represents the rectified sEMG signal of muscle m at the $i^{th}$ sample; $N_{m}$ represents the total number of samples of muscle m included in the analysis. $Contrib_{m}$ represents the relative contribution rate of muscle m, and the denominator corresponds to the summed time-normalized sEMG of all 16 analyzed muscles. The inclusion of this feature is because it indicates the proportional output of the muscles during walking, highlighting the primary load-bearing muscles. Higher relative contribution rates suggest increased stress on specific muscle groups, aiding in identifying those at increased risk of fatigue and falls due to excessive load. After converting the signals from time domain to frequency domain by the Fast Fourier Transform (FFT), MF and MPF can be determined as shown in Equations 2,3:

$$\overset{k^{*}}{\underset{k=0}{\sum }}PSD\left(f_{k}\right)=\frac{1}{2}\overset{K}{\underset{k=0}{\sum }}PSD\left(f_{k}\right),\;MF=f_{k^{*}}$$ (2)

$$MPF=\frac{\overset{K}{\underset{k=0}{\sum }}f_{k}\;PSD\left(f_{k}\right)}{\overset{K}{\underset{k=0}{\sum }}PSD\left(f_{k}\right)}$$ (3)

where $PSD\left(f_{k}\right)$ represents the power spectral density of the sEMG signals; $k^{*}$ represents the frequency index at which the cumulative spectral power reaches half of the total power; K denotes the total number of discrete frequency bins considered in the power spectral density. The incorporation of MF and MPF reflects their ability to capture the frequency distribution of muscle activation, providing information on muscle recruitment patterns and fatigue. In this study, some commonly used metrics, such as peak value and cumulative impulse, are excluded from the final model. Although they measure muscle force output, experts believe that they do not show the relative contribution of each muscle during the gait cycle or provide a standardized basis for comparison in whole-body movement. Hence, finally the sEMG features in our model are contribution, MF, and MPF for each select muscles and different muscle groups (listed in Table A2 in the Appendix).

Dynamic plantar pressure signals are collected as time series data to study the displacement of the center of mass, which consists of shifts between the left and right feet and between the front and back of the feet. Based on the points shown in Figure 3, data from the eight sensor points on the left foot are used to calculate the dynamic loading of the left foot, while data from the eight sensor points on the right foot are used for the dynamic loading of the right foot. For the forefoot, the dynamic load is calculated using data from five pressure points: the big toe, the first metatarsal, the 2nd-3rd metatarsals, the 4th-5th metatarsals and the medial arch. For the rearfoot, dynamic loading is calculated using data from three pressure points: medial, posterior, and lateral heel. The cumulative pressure impulses for the left and right feet are calculated as

$$\begin{array}{ll} X_{L} & =\overset{T}{\underset{j=1}{\sum }}\left(\underset{i\in L}{\sum }P_{i}\left(t_{j}\right)\;A_{i}\right)\Delta t,\\ X_{R} & =\overset{T}{\underset{j=1}{\sum }}\left(\underset{i\in R}{\sum }P_{i}\left(t_{j}\right)\;A_{i}\right)\Delta t; \end{array}$$

while forefoot and hindfoot is calculated as:

$$\begin{array}{ll} X_{F} & =\overset{T}{\underset{j=1}{\sum }}\left(\underset{i\in F}{\sum }P_{i}\left(t_{j}\right)\;A_{i}\right)\Delta t,\\ X_{B} & =\overset{T}{\underset{j=1}{\sum }}\left(\underset{i\in B}{\sum }P_{i}\left(t_{j}\right)\;A_{i}\right)\Delta t, \end{array}$$

where $P_{i}\left(t_{j}\right)$ represents the pressure data at the i -th sensor point at time $t_{j}$ ; $A_{i}$ stands for the area of the i -th sensor point; j indexes discrete sampling instants; and $\sum_{i\in \Omega }P_{i}\left(t_{j}\right)$ equals the finite sum of the pressure data within the zone Ω . Δt is the sampling interval, and T is the total number of samples. L and R denote the sets of all eight snesor points on the left and right foot, respectively, while F and B denote the sets of forefoot (10 points) and rearfoot (6 points) sensors from both feet. Ultimately, by calculating $X_{L},X_{R},X_{F},$ and $X_{B}$ from the 16 sensor points, we obtain the cumulative pressure and percentage contribution of the left and right feet, as well as the forefoot and hindfoot. A description of the characteristics related to the plantar pressure distribution is provided in Table A3 in Appendix A.

In sum, 76 signal-derived features (72 sEMG features and four plantar-pressure features) were extracted before feature selection. Together with five demographic features, the final input space comprised 81 input features.

### 3.2 Statistical analysis (ANCOVA)

According to Section 2.1, participants were divided into a control group (Class 0) and an experimental group (Class 1). A Chi-squared test found no significant difference in sex (p=0.423) . Independent-sample t tests indicated significant group differences in age, height, and weight ( p<0.001 , p<0.001 , and p=0.006 ), while BMI showed no difference (p=0.468) . These results suggest that demographic factors differ between groups and must therefore be controlled in subsequent analyses (Table 1).

**TABLE 1:** Summary of t-tests for basic information.

Basic information	p -value
Age	**0.000**
Height	**0.000**
Weight	**0.006**
BMI	0.468

Bold values indicate statistically significant or marginally significant differences (p < 0.10).

To account for these demographic differences, ANCOVA was performed on all sEMG and plantar-pressure features, controlling for age, height, weight, and BMI (Table 2). Several frequency-domain sEMG measures (MF and MPF) showed significant group effects, predominantly in left-limb muscles. Specifically, MF values were significantly different for Tibialis Anterior (L), Peroneus Longus (L), Medial Gastrocnemius (L), Vastus Medialis (L), Rectus Femoris (L), and Biceps Femoris (L) (p<0.05) . MPF values also showed significant or marginal differences in Peroneus Longus (L), Vastus Medialis (L), Rectus Femoris (L), and Biceps Femoris (L). These findings indicate that older adults at higher fall risk tend to exhibit altered neuromuscular frequency characteristics particularly on the left side, consistent across both individual muscles and broader muscle groups (Table 3).

**TABLE 2:** Summary of ANCOVA results for plantar pressure data (group-effect p -values adjusted for age, height, weight, and BMI).

Plantar pressure	p -value (group)
Left foot	0.635
Right foot	0.927
Front foot	0.110
Back foot	**0.014**

Bold values indicate statistically significant or marginally significant differences (p < 0.10).

**TABLE 3:** Summary of ANCOVA results for individual muscles and muscle groups (group-effect p -values adjusted for age, height, weight, and BMI).

sEMG features	Contribution	MF	MPF
Muscles			
Tibialis anterior, R	0.266	0.758	0.574
Peroneus longus, R	**0.091**	0.155	0.148
Lateral gastrocnemius, R	0.664	0.299	0.667
Medial gastrocnemius, R	0.809	0.647	0.754
Tibialis anterior, L	0.149	**0.039**	0.397
Peroneus longus, L	0.977	**0.011**	**0.028**
Lateral gastrocnemius, L	0.508	0.155	0.345
Medial gastrocnemius, L	0.732	**0.019**	0.148
Vastus medialis, R	0.546	0.870	0.629
Rectus femoris, R	0.932	0.253	0.317
Vastus lateralis, R	0.746	0.161	0.179
Biceps femoris, R	0.929	0.472	0.542
Vastus medialis, L	0.972	**0.018**	**0.021**
Rectus femoris, L	0.583	**0.049**	**0.063**
Vastus lateralis, L	0.972	0.168	0.136
Biceps femoris, L	0.290	**0.009**	**0.038**
Muscle groups			
Thigh, L	0.676	**0.010**	**0.022**
Lower leg, L	0.755	**0.009**	**0.059**
Thigh, R	0.614	0.292	0.294
Lower leg, R	0.439	0.414	0.782
Left leg	0.852	**0.007**	**0.018**
Right leg	0.852	0.345	0.417
Thighs	0.453	**0.071**	0.249
Lower legs	0.453	**0.056**	**0.081**

Bold values indicate statistically significant or marginally significant differences (p < 0.10).

At the muscle-group level, the left thigh, left lower leg, and the overall left leg showed significant group effects in MF and MPF, with additional marginal effects observed for bilateral thigh and lower-leg groupings. In contrast, Contribution features showed generally weak group effects, with Peroneus Longus (R) being the only muscle approaching marginal significance (p=0.091) .

For plantar-pressure features, the back foot exhibited a significant group difference (p=0.014) , whereas the front foot showed a close to the marginal trend (p=0.110) , suggesting potential alterations in foot-loading patterns during gait in individuals with elevated fall risk.

It is important to note that a lack of statistical significance in ANCOVA does not imply that a feature is unimportant for fall-risk prediction. Interactions among factors such as muscle activation, movement strategies, and plantar pressure may collectively contribute to classification performance even when individual effects appear weak in isolation. Moreover, several features showing marginal significance (0.05<p<0.10) may reflect meaningful physiological differences that did not reach full statistical significance due to the limited sample size; their true effects may become clearer as more participants are included in future studies.

### 3.3 Dimension reduction

With numerous features and a limited number of participants, it is essential to apply dimensionality reduction techniques prior to model training. This step mitigates the risk of overfitting, improves model generalizability, and allows for more robust analysis given the constraints of the dataset.

#### 3.3.1 Principal component analysis

Principal Component Analysis (PCA) is a widely used statistical method for dimensionality reduction that reveals the core structure of the data by mapping high-dimensional data into a lower-dimensional subspace while preserving as much variance as possible. By identifying the principal components, representing the maximum-variance directions in the data, PCA facilitates this reduction. These components, which are the eigenvectors of the data’s covariance matrix, are orthogonal and maximize data variance. By selecting the first few principal components, PCA retains significant variability while ignoring noise and less important features, thus providing a measure of explained variance as an indicator of retained information (Wang et al., 2020; Fernández et al., 2018).

The PCA process involves several key steps: calculating the covariance matrix, extracting eigenvalues and eigenvectors, selecting principal components, and transforming the data into a new space. The covariance matrix, which describes the relationships between variables, is computed as follows:

$$Cov\left(X\right)=\frac{1}{n-1}X^{T}X,$$

where X is the centered data matrix and n is the number of observations. The eigenvectors and eigenvalues indicate the principal axes of variation and the corresponding spread of the data. Typically, the eigenvectors with the largest eigenvalues are selected as the main components, determined by the cumulative contribution of the eigenvalues, often aiming for a threshold of 80% of total variance. Finally, the original data matrix X is transformed into the principal component space by:

Y=XV,

where V is the matrix of selected eigenvectors. This transformation not only reduces the dimensionality but also uncovers the intrinsic structure of the data, laying the groundwork for further analysis.

#### 3.3.2 Linear discriminant analysis

Linear Discriminant Analysis (LDA) aims to find an optimal linear projection that maximally separates data from different classes. LDA projects the data into a lower-dimensional space while maximizing the distance between classes and minimizing the distance within each class. This process not only reduces the dimensionality but also enhances the classification performance by constructing a linear decision boundary that effectively distinguishes between classes. LDA achieves this using the within-class scatter matrix $S_{w}$ and the between-class scatter matrix $S_{b}$ .

The within-class scatter matrix $S_{w}$ measures the variance of the data points within a class relative to the class mean, defined for each class c as:

$$S_{w}=\underset{x\in c}{\sum }\left(x-m_{c}\right){\left(x-m_{c}\right)}^{T}.$$

The total within-class scatter matrix is the sum across all classes:

$$S_{w}=\sum S_{wc}.$$

Conversely, the between-class scatter matrix $S_{b}$ captures the differences between the class means:

$$S_{b}=\sum N_{c}\left(m_{c}-m\right){\left(m_{c}-m\right)}^{T}.$$

To identify a linear projection ω that maximizes $S_{b}$ while minimizing $S_{w}$ , LDA maximizes the ratio:

$$J\left(\omega \right)=\frac{\omega^{T}S_{b}\omega }{\omega^{T}S_{w}\omega }.$$

Using the Lagrange multiplier method, the optimal ω corresponds to the eigenvector associated with the highest eigenvalue of $S_{w}^{-1}S_{b}$ . Once determined, this projection direction enables the mapping of data points to a lower-dimensional space, effectively completing the dimensionality reduction process.

### 3.4 Forecasting models

#### 3.4.1 Support vector machine classifier

Support Vector Machines (SVMs) are a powerful classification method that aims to partition data into two categories by constructing an optimal hyperplane. SVMs are often used due to their effectiveness with small sample sizes (Güler and Ker, 2005; Gokgoz and Subasi, 2014). In a two-dimensional space, this hyperplane is represented by a line, while in higher-dimensional spaces, it is a plane or a more complex hyperplane. The primary objective of SVM is to find a hyperplane that can correctly classify all training samples while maximizing the distance between it and the closest training samples. The hyperplane that maximizes the distance is considered optimal. When the data are not linearly separable, SVM introduces kernel functions to map the original feature space to a higher-dimensional space, enabling the identification of a hyperplane that can linearly separate the data. In this study, the gamma parameter was set to auto, which assigns gamma as the inverse of the number of features, allowing the model to automatically adjust this essential parameter for the kernel function. The kernel used was the Radial Basis Function (RBF) kernel, which is commonly used in SVM models.

#### 3.4.2 Random forest classifier

The Random Forest (RF) classifier uses an ensemble of decision trees to improve the accuracy and generalization of prediction. One of the primary advantages of using RF in sEMG applications is its ability to manage high-dimensional data and mitigate overfitting through ensemble learning. For example, (Du et al., 2017), demonstrated that RF, when combined with a manually designed set of features from sEMG signals, can achieve commendable performance in gesture recognition tasks (Du et al., 2017). It has proven to be an effective method and has been widely applied in numerous studies involving the processing of sEMG signals (Shen et al., 2019; Li et al., 2019; Karthick et al., 2018). The RF classifier relies on two main principals: bootstrap sampling and random feature selection. Bootstrap sampling generates diverse training subsets by drawing multiple random samples with replacement from the original dataset. Random feature selection further diversifies the model by randomly choosing a subset of features at each decision node rather than using all features. The RF training process involves several steps to build each tree. First, bootstrap sampling creates a training dataset for each tree. Then, at each node, a random subset of features is selected and the optimal splitting feature is chosen based on criteria like information gain or Gini impurity. This recursive process continues until specific stopping conditions are met, such as a minimum number of samples per node or a maximum tree depth. For classification, each tree independently classifies the input and the final result is determined by majority vote. In this study, the RF classifier was configured with 100 trees (n_estimators) and used Gini impurity (gini) as the criterion for evaluating splits. The default setting for max_features was applied, allowing consideration of all features at each split.

#### 3.4.3 Extreme gradient boosting classifier

The Gradient Boosting algorithm incrementally adds weak learners, typically decision trees, to correct residual errors from previous iterations, progressively improving model accuracy. XGBoost (Extreme Gradient Boosting) is an enhanced version of traditional Gradient Boosting, introducing optimizations such as regularization and computational efficiency that significantly improve performance and broaden the model’s applicability. These advancements have established XGBoost as a highly effective machine learning technique for sEMG signal classification, offering remarkable accuracy and adaptability in various applications (Ghaderi et al., 2022; Chen et al., 2023; Song et al., 2023). XGBoost operates within the Gradient Boosting framework, constructing decision trees in each iteration to address prediction errors from prior rounds. A notable feature is its use of randomness in feature selection, where a subset of features is randomly chosen for each split rather than searching across all features. This approach reduces computational cost and promotes model diversity, enhancing generalization. In this study, the number of trees for XGBoost, specified by n_estimators, was set to 100, aligning its complexity with that of the Random Forest model.

### 3.5 Performance metrics

The model performances are evaluated via four commonly employed metrics: Accuracy, Precision, Recall, and F1 score. These metrics assess the comparison between the predictions of the model and the actual values, as shown in Table 4. The confusion matrix includes True Positive (TP), False Positive (FP), True Negative (TN), and False Negative (FN).

**TABLE 4:** Confusion matrix for classification evaluation.

Confusion matrix		Truth	
		Positive	Negative
Prediction	Positive	TP	FP
	Negative	FN	TN

Accuracy offers a comprehensive measure of a model’s overall performance, while Precision and Recall provide deeper insights into the quality and completeness of predictions for the positive class. Precision evaluates the correctness of positive predictions, whereas Recall assesses the model’s ability to identify all instances of the positive class. The definitions for Accuracy, Precision, and Recall are given in Equations 4–6:

$$\text{Accuracy}=\frac{TP+TN}{TP+TN+FP+FN}$$ (4)

$$\text{Precision}=\frac{TP}{TP+FP}$$ (5)

$$\text{Recall}=\frac{TP}{TP+FN}$$ (6)

The F1 score is the harmonic mean of precision and recall, balancing the two metrics. It is used when both precision and recall are important to evaluate. The definition of the F1 score is given in Equation 7:

$$\text{F}1\text{ score}=2\times \frac{\text{Precision}\times \text{Recall}}{\text{Precision}+\text{Recall}}$$ (7)

## 4 Results

### 4.1 Computing environment

The algorithm runs on a machine equipped with an Intel(R) Core(TM) i5-8250U CPU at 1.60 GHz, which features eight logical cores, providing substantial processing power for multitasking and data-intensive operations. For graphics processing and visualization tasks, the system utilizes dual graphics cards: an Intel(R) HD Graphics 620 and an NVIDIA GeForce MX150. The system operates on Windows 11, which offers a stable and secure environment for deploying web applications.

### 4.2 Data source

**FIGURE 4:**
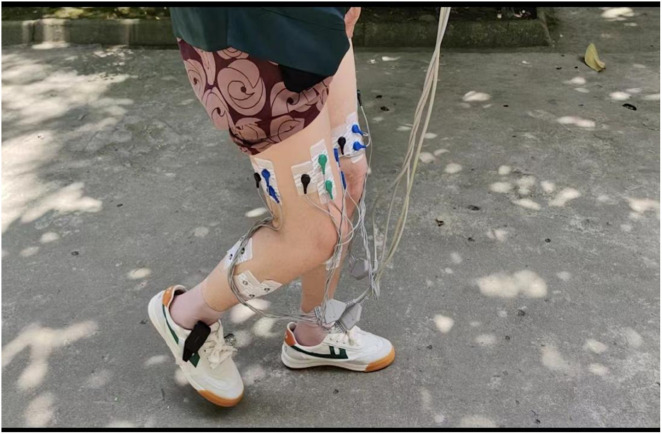
Sensor placement on a participant during walking.

In this study, 94 subjects who meet the inclusion criteria are recruited from the outpatient clinic of West China Hospital, Sichuan University (personal information summarized in Table 5). A West China Hospital physician took charge of this data collection that lasted from February 2023 to October 2023. As illustrated during the data collection process, participants wore shoes equipped with plantar pressure sensors throughout the duration. sEMG sensors were placed in key muscle groups of the lower extremities to acquire data during walking. Figure 4 provides a view of the placement of the sensors in the participants, showing the locations where the sensors were attached.

**TABLE 5:** Summary statistics for participants.

Variable	Mean	Std dev	Max	Min
Age (years)	69.64	10.0	95.0	60.0
Height (cm)	157.76	9.0	176.0	135.0
Weight (kg)	59.22	9.99	90.0	40.0
BMI (kg/m^2^)	23.84	3.99	43.90	15.76

### 4.3 Classification performance

Three different classification models, Support Vector Machine (SVM), Random Forest (RF), and XGBoost, are applied to the dataset. The Leave-One-Out Cross-Validation (LOOCV) method is used to evaluate classification performance. LOOCV is particularly suited for small datasets with a large number of features. Previous studies have highlighted the necessity of using LOOCV in training electromyography (EMG) models, especially when sample sizes range from 26 to 135 cases (Wong, 2015; Zhao et al., 2022; Fricke et al., 2021). LOOCV uses each individual sample from the dataset as a test set, while the remaining samples are used as the training set. Specifically, if there are N samples, LOOCV conducts N rounds of training and testing, each round selecting one sample as the test set and the remaining N−1 samples as the training set. The optimal training hyperparameters of three models obtained through experimentation are summarized in Table 6.

**TABLE 6:** Hyperparameter settings.

Model	Hyperparameter settings
SVM	kernel = “rbf”, gamma = “auto”
RF	n_estimators = 100; criterion = “gini”; max_features = “sqrt”
XGB	objective = “binary: logistic”; n_estimators = 100

**FIGURE 5:**
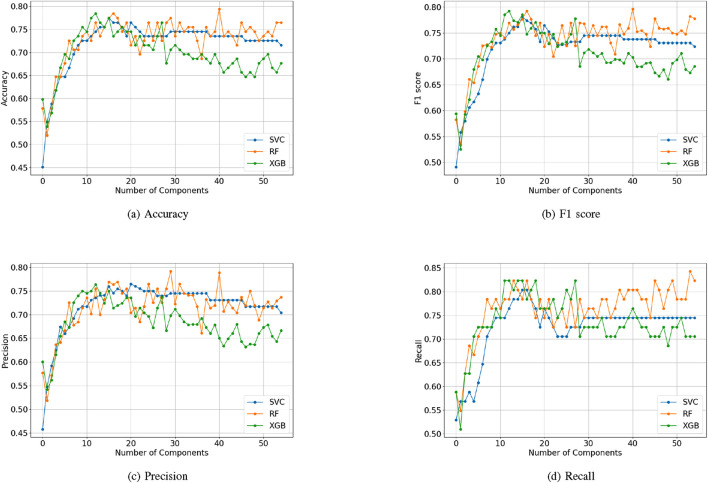
Performance of models with PCA: **(a)** Precision. **(b)** Recall. **(c)** Accuracy. **(d)** F1 score.

In the case of PCA, Figure 5 illustrates how Accuracy (Figure 5a), F1 score (Figure 5b), Precision (Figure 5c) and Recall (Figure 5d) change as the number of principal components increases for different models. Figure 5a shows that the Accuracy of all three models increases as the number of principal components grows, and then becomes stable as the number of components exceeds 10. The SVM model attained an optimal accuracy of 0.77 with 16 principal components, while the RF and XGB models achieved a maximum accuracy of 0.79 with 18 and 13 principal components, respectively. Similar patterns can also be observed for F1 score, Precision and Recall with the best performance occurring between 10 and 20 components involved. After reaching the peaks, the metrics for SVM and RF slightly declined but remained stable as the number of dimensions increased. For XGB, there shows a downward trend as the principal component increases. It can be observed that increasing the number of components does not always lead to better classification performance, with the best performance observed in the 10- to 20-dimensional range. Furthermore, SVM and RF provided comparable performance across all metrics, while XGB exhibited a significant decline in performance as dimensionality increased.

**FIGURE 6:**
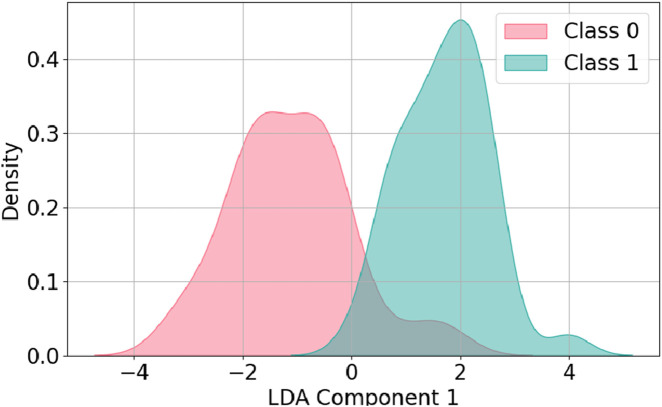
Data distribution after LDA dimension reduction.

In the case of LDA, Figure 6 shows the probability density distribution of the dataset after projection onto a single discriminant component. The plot clearly indicates a strong separation between Class 0 and Class 1, with minimal overlap across most of the range. This separation suggests that LDA has successfully maximized the class distinction in the projected space. The ability to achieve such separation highlights LDA’s strong discriminative power, ensuring that instances from different classes remain well-separated along this dimension. This result confirms the effectiveness of LDA in improving class distinction and improving classification performance.

The predictive performance of different models is summarized in Table 7. The original data refers to the dataset without dimensionality reduction. The results show that PCA improves performance across all metrics, though not as effectively as LDA. These findings indicate that the application of a dimensionality reduction technique is essential to achieve better predictive performance. For each machine learning model, the highest accuracy, precision, recall, and F1 score are achieved when LDA is applied. Among all models, SVM with LDA achieves the best overall performance, followed by XGBoost with LDA and RF with LDA. These results align with the earlier observation in Figure 6, where LDA effectively separates the two target classes.

**TABLE 7:** Results of LOOCV with different models.

Method	Accuracy	Precision	Recall	F1 score
SVM + original data	0.71	0.70	0.74	0.72
SVM + LDA	**0.88**	**0.92**	**0.85**	**0.87**
SVM + PCA	0.77	0.71	0.83	0.77
RF + original data	0.74	0.70	0.80	0.75
RF + LDA	**0.86**	**0.89**	**0.82**	**0.85**
RF + PCA	0.79	0.78	0.80	0.79
XGB + original data	0.78	0.79	0.78	0.78
XGB + LDA	**0.88**	**0.90**	**0.83**	**0.87**
XGB + PCA	0.79	0.82	0.76	0.79

Bold values indicate the highest value(s) in each performance metric.

### 4.4 Clinical data testing

To further validate the effectiveness of the predictive framework, we collect an additional clinical validation set from West China Hospital. This dataset consists of 10 clinical cases collected between November 2023 and February 2024. The trained models are applied to this new dataset, and the prediction Accuracy are presented in Table 8. It shows that the models trained on the original data do not perform well. Both PCA and LDA improve the results, and LDA provides greater enhancement than PCA. Among all models, LDA combined with SVM and RF achieves the highest accuracy in this set of tests. These findings confirm that LDA is more effective than PCA in reducing dimensionality and are consistent with the previous results.

**TABLE 8:** Accuracy of models for the validation set.

Model	Original data	LDA	PCA
SVM	0.7	0.8	0.7
RF	0.6	0.8	0.7
XGB	0.7	0.7	0.6

## 5 Discussions

This study demonstrates, for the first time, that the risk of falling can be predicted using a well-designed wearable system that combines sEMG and plantar pressure sensors. The wearable devices used in this research are both affordable and highly portable, making them suitable for use in both high-income and resource-limited communities. One key advantage of this approach is that it does not require invasive procedures. In addition, the devices do not need to be worn continuously for long-term monitoring in daily life. Instead, they collect data over a short period for screening purposes, eliminating the inconvenience of constant sensor attachment.

Based on the collected sEMG and plantar pressure data, we have identified several important clinically significant findings that can serve as key indicators to predict the risk of falling in older adults. Recall Tables 2, 3, between Class 0 and Class 1, MF shows significant differences in eight individual muscles, seven of them located in the left leg. For MPF, six muscles show significant differences in the left leg. That is, older adults with a high risk of falling tend to have more frequent muscle activity in their left leg. For plantar pressure data, there is a difference in back foot pressure between Class 0 and Class 1. This finding indicates that the distribution of plantar pressure during walking differs significantly between the two groups, where older adults with a high risk of falling tend to put their weight on the back of the foot. These results deserve further exploration by clinical researchers to deepen our understanding of the risk of falls in older adults.

The SVM, RF, and XGBoost classifiers are widely used classification methods, each with distinct strengths and suitable application scenarios. SVM is advantageous for handling high-dimensional datasets with clear class boundaries. RF performs well on noisy data with complex feature interactions. XGBoost is suitable for complex datasets where high prediction accuracy is required. In this study, we ultimately chose the LDA + SVM combination because when data are reduced to one dimension by LDA and show good linear separability, SVM is an effective choice, as it can construct a maximum-margin linear boundary that supports accurate and stable classification. In contrast, PCA did not perform well in our study, possibly due to the limited sample size with large data dimensions. In future work, as more participants are collected, we will continue to evaluate the effectiveness of PCA in dimensionality reduction.

Table 9 provides an overview of representative multimodal EMG-based systems and summarizes their key sensing configurations, target populations, and experimental settings. In addition to this descriptive comparison, a more detailed analysis highlights several practical advantages of the proposed dual-modality system over existing EMG-based multimodal approaches. In terms of applicability, prior systems such as those of Xi et al. (2020) and Cheng et al. (2013) were evaluated primarily in healthy young adults and relied on simulated or laboratory-induced falls, limiting their relevance to real-world geriatric populations. By contrast, the present study evaluates fall-risk prediction during natural walking in a clinical cohort of older adults, making the findings directly applicable to the target population.

**TABLE 9:** Comparison of multimodal wearable systems for fall detection and fall-risk assessment.

Study	Modalities	Population	Task/Objective	Limitations compared to this work
Xi et al. (2020)	4-ch sEMG +3 plantar-pressure channels	12 healthy young adults (23–27 years)	Classification of postures, gait activities, and experimentally induced falls	Not older adults; no fall-risk stratification; small sample; controlled experiments only
Cheng et al. (2013)	sEMG + trunk/thigh accelerometers	10 healthy adults (22–26 years)	Daily activity recognition and simulated fall detection using HMM-based framework	Not elderly; simulated falls; limited ecological validity; no plantar-loading information
Siwadamrongpong et al. (2022)	sEMG + hip-mounted IMU	1 healthy young male (24 years)	Prototype system for fall detection and early fall prediction	Extremely small sample; not elderly; no gait-pressure data; feasibility study only
da Silva et al. (2024)	sEMG + dynamometry (maximal isometric contractions)	30 older adults	ML-based prediction of BBS scores and fall risk	Non-wearable equipment; no walking data; no plantar-pressure information
This Study	16-ch sEMG + 4-region plantar-pressure insoles	Older adults (clinical cohort)	Fall-risk prediction during natural walking + model validation on independent dataset	Portable, interpretable neuromuscular + plantar-loading integration; clinically deployable

Regarding ease of use, methods that incorporate IMUs or multi-site accelerometers often require numerous body-mounted sensors and precise placement (Cheng et al., 2013; Siwadamrongpong et al., 2022), and dynamometry-based assessment requires non-portable laboratory equipment (da Silva et al., 2024). The proposed wearable system uses only surface-mounted electrodes and pressure insoles, requires minimal setup time, and enables short-duration screening without continuous monitoring, making it more suitable for deployment in community and clinical environments.

In terms of accuracy, existing multimodal studies have demonstrated strong performance for fall-event detection, but few have achieved clinically meaningful fall-risk prediction. The present framework combines physiologically interpretable features—neuromuscular activation patterns and plantar-loading distributions—with a lightweight LDA + SVM classifier, achieving robust discrimination between low- and high-risk individuals while maintaining low computational cost. This balance of accuracy, interpretability, and practicality distinguishes the proposed system from prior multimodal designs and strengthens its potential for use in routine fall-risk assessment workflows.

This study has some limitations that should be acknowledged. First, although the retrospective study design allows the use of existing clinical data, it inevitably carries a risk of bias, as patient selection and data collection are not ideally randomized or controlled. This may lead to unbalanced group characteristics or confounding variables that influence the results. In addition, the current data size remains limited. More participant data are needed to further validate the robustness of the proposed predictive model. Furthermore, this study does not include prior biomechanical research, such as gait analysis or muscle activation patterns, which could provide a stronger theoretical foundation to interpret clinical findings. These issues should be addressed and improved in future work.

## 6 Conclusion

This study successfully demonstrates the feasibility of fall-risk prediction using an integrated framework combining wearable data acquisition and AI-based offline analysis. Portable, non-invasive devices are used only during screening, enhancing patient compliance and practicality in various settings. Statistical analyzes confirm that the features of the sEMG and plantar pressure sensors significantly impact the risk of falls. By integrating dimensionality reduction and machine learning methods, the proposed framework effectively predicts fall risk, allowing for prompt interventions to prevent falls. This system performs well in a clinical validation study, shows its potential for broader adoption in the assessment of the risk of falls. In this study, model training and evaluation were performed offline on pre-recorded wearable-sensor data, rather than through real-time on-body deployment.

## Data Availability

The original contributions presented in the study are included in the article/supplementary material, further inquiries can be directed to the corresponding authors.

## Appendix A

**TABLE A1:** Basic information details.

Index	Feature	Description
1	Gender	Male/female
2	Age	Age of the subject
3	Height	Height of the subject (cm)
4	Weight	Weight of the subject (kg)
5	BMI	Body mass index

**TABLE A2:** Muscle groups details.

Index	Muscle group
1	Left thigh
2	Left lower leg
3	Right thigh
4	Right lower leg
5	Left leg
6	Right leg
7	Thighs (both)
8	Lower legs (both)

**TABLE A3:** Plantar pressure distribution details.

Index	Feature	Description
1	$X_{L}$	Left foot pressure distribution (%)
2	$X_{R}$	Right foot pressure distribution (%)
3	$X_{F}$	Forefoot pressure distribution (%)
4	$X_{B}$	Rearfoot pressure distribution (%)
